# Bactericidal activity of hexylresorcinol lozenges against oropharyngeal organisms associated with acute sore throat

**DOI:** 10.1186/s13104-020-04954-1

**Published:** 2020-02-24

**Authors:** Derek Matthews, Oluwajoba Adegoke, Adrian Shephard

**Affiliations:** 1 Reckitt Benckiser Healthcare International Ltd, 103–105 Bath Road, Slough, Berkshire, SL1 3UH UK; 2 Reckitt Benckiser Healthcare UK Ltd, Dansom Lane, Hull, HU8 7DS UK

**Keywords:** Antibacterial agents, Antiseptic, Bacterial infection, Bactericidal, Pharyngitis, Sore throat

## Abstract

**Objective:**

For the majority of people with acute sore throat, over-the-counter treatments represent the primary option for symptomatic relief. This study evaluated the in vitro bactericidal activity of lozenges containing the antiseptic hexylresorcinol against five bacteria associated with acute sore throat: *Staphylococcus aureus*, *Streptococcus pyogenes, Moraxella catarrhalis*, *Haemophilus influenzae* and *Fusobacterium necrophorum.*

**Results:**

Hexylresorcinol 2.4 mg lozenges were dissolved into 5 mL of artificial saliva medium. Inoculum cultures were prepared in triplicate for each test organism to give an approximate population of 10^8^ colony-forming units (cfu)/mL. Bactericidal activity was measured by log reduction in cfu. Greater than 3log_10_ reductions in cfu were observed at 1 min after dissolved hexylresorcinol lozenges were added to *S. aureus* (log_10_ reduction cfu/mL ± standard deviation, 3.3 ± 0.2), *M. catarrhalis* (4.7 ± 0.4), *H. influenzae* (5.8 ± 0.4) and *F.* *necrophorum* (4.5 ± 0.2) and by 5 min for *S. pyogenes* (4.3 ± 0.4). Hexylresorcinol lozenges achieved a > 99.9% reduction in cfu against all tested organisms within 5 min, which is consistent with the duration for a lozenge to dissolve in the mouth. In conclusion, in vitro data indicate that hexylresorcinol lozenges offer rapid bactericidal activity against organisms implicated in acute sore throat.

## Introduction

Acute sore throat is a common symptom of an upper respiratory tract infection (URTI), associated with inflammation of the pharynx, tonsils or nasopharynx [1]. The most frequent cause of acute sore throat is a viral infection, responsible for up to 80% of cases in adults [2]. Bacterial infections are estimated to cause 5–15% of acute sore throat cases in adults [3–5]. *Streptococcus pyogenes* (also known as group A β-hemolytic *Streptococcus* or GABHS) is the most common bacterial cause of acute sore throat [3, 5], although other bacterial species have been implicated, including *Staphylococcus aureus* [6], *Moraxella catarrhalis* [7], *Haemophilus influenzae* [8] and *Fusobacterium necrophorum* [9].

Antibiotics continue to be overprescribed for acute sore throat and are often unnecessary and ineffective in this setting [10], contributing to the growing problem of antibiotic resistance [11]. Even when the cause of sore throat is bacterial, in most cases it will be self-limiting and improve without the need for antibiotics [3]. For most individuals, over-the-counter treatments, such as lozenges, represent the primary option for relief from the symptoms of acute sore throat [12]. Lozenges containing the antiseptic hexylresorcinol significantly reduced the symptoms of acute sore throat over a 2-h study period in a placebo-controlled trial [13]. Furthermore, a concentration-dependent numbing effect with hexylresorcinol lozenges has been reported in healthy volunteers [14]. These effects are likely in part due to its local anesthetic activity, achieved through blocking voltage-gated neuronal sodium channels [15]. Hexylresorcinol has also demonstrated antiviral effects against species associated with URTIs [16] or known to cause acute sore throat [17].

In vitro studies have found that hexylresorcinol has antibacterial activity against a range of species when in solution and when embedded in biopolymer composite films [18, 19]. The antibacterial effects of hexylresorcinol in vivo may be mediated through several mechanisms including reducing bacterial adherence to the pharynx, inhibiting bacterial biofilm formation, disrupting bacterial cell chain formation, and modifying cell surface hydrophobicity [20]. However, no published studies have addressed whether hexylresorcinol lozenges have antibacterial activity against organisms implicated in acute sore throat.

This study determined the in vitro bactericidal activity of hexylresorcinol lozenges against a range of medically relevant oropharyngeal organisms associated with acute sore throat.

## Main text

### Methods

#### Test samples

Hexylresorcinol 2.4 mg lozenges (Strepsils Extra Honey and Lemon lozenges; Reckitt Benckiser, Slough, UK) were dissolved at 44 ± 1 ℃ into 5 mL of artificial saliva medium as described previously [21].

#### Test organisms

*S. aureus* (NCTC7445, Public Health England, Salisbury, UK), *S. pyogenes* (NCTC12696, Public Health England), *M. catarrhalis* (NCTC3622, Public Health England), *H. influenzae* (NCTC4842, Public Health England) and *F. necrophorum* (NCTC12238, Public Health England) were cultured as described previously [21].

#### Bactericidal assay

The bactericidal assay was performed using a method similar to that described previously [21]. Briefly, inoculum suspensions prepared in triplicate for each test organism, at approximately 10^8^ colony-forming units (cfu)/mL in saline, were mixed with hexylresorcinol test sample (4.9 mL). Bactericidal activity was assayed after 1-, 5-, 10- and 30-min exposure times by combining sample/inocula mixture (1 mL) with neutralizing diluent (9 mL). Serially-diluted solutions were incubated on suitable agar medium for at least 3 days. Bactericidal activity was also assayed at the 30-min time point for inoculum cultures (0.1 mL) for each test organism mixed with a positive control sample of artificial saliva medium (4.9 mL). Mean log reduction (in cfu/mL) for test samples was calculated for each organism and time point (average of three triplicates) relative to test controls.

## Results

**Fig. 1:**
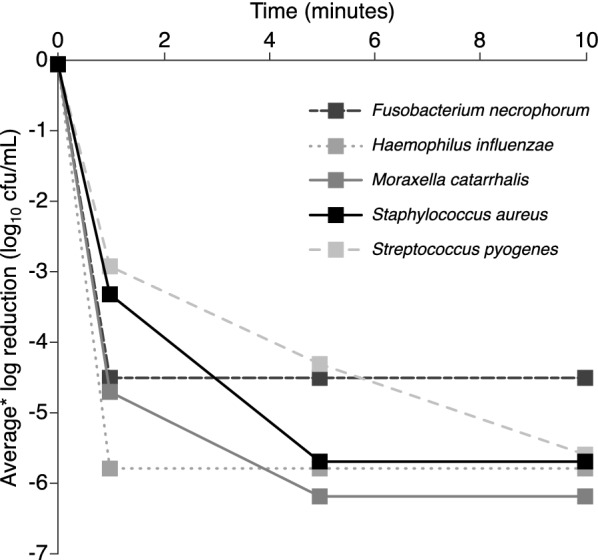
Bactericidal activity of hexylresorcinol lozenges. Bactericidal activity, defined as a decrease in bacterial count (log_10_ cfu/mL), was measured against five common oropharyngeal organisms over a 30-min period. Only activity measurements up to 10 min are shown. *Average of the three test replicates. *cfu* colony-forming units

Test control counts demonstrated that the test method and media did not affect the survival of the organisms. Following test sample inoculation, evidence of bactericidal activity was recorded at the 1-min time point for all organisms (Table 1, Fig. 1). For *S. aureus*, *M.* *catarrhalis*, *H. influenzae* and *F. necrophorum*, the decrease at 1 min exceeded 3log_10_ (99.9% reduction) (Table 1). For *S. pyogenes*, a 2.9log_10_ reduction was seen at 1 min and a greater than 3log_10_ reduction was recorded by 5 min (Table 1). For all test organisms, the lower limit of detection in the bactericidal activity assay was reached at the 30-min time point (Table 1).

**Table 1:** Bactericidal activity of hexylresorcinol lozenges

Challenge organism	Test control count* (log_10_ cfu/mL ± SD)	Contact time (min)			
		1	5	10	30
		Average^a^ log reduction (log_10_ cfu/mL ± SD)			
*Staphylococcus aureus*	6.7 ± 0.1	3.3 ± 0.2	5.7 ± 0.2	5.7 ± 0.2	5.7 ± 0.2
*Streptococcus pyogenes*	6.6 ± 0.1	2.9 ± 0.2	4.3 ± 0.4	5.6 ± 0.1	5.6 ± 0.1
*Moraxella catarrhalis*	7.2 ± 0.1	4.7 ± 0.4	6.2 ± 0.1	6.2 ± 0.1	6.2 ± 0.1
*Haemophilus influenzae*	6.8 ± 0.4	5.8 ± 0.4	5.8 ± 0.4	5.8 ± 0.4	5.8 ± 0.4
*Fusobacterium necrophorum*	5.5 ± 0.2	4.5 ± 0.2	4.5 ± 0.2	4.5 ± 0.2	4.5 ± 0.2

Bactericidal activity, defined as a decrease in bacterial count (log_10_ cfu/mL)

*cfu* colony-forming units, *SD* standard deviation

*Average of the three test replicates

## Discussion

Hexylresorcinol lozenges demonstrated rapid antibacterial activity against a broad range of organisms implicated in acute sore throat, including Gram-positive species (*S. aureus* and *S. pyogenes*) and Gram-negative species (*M. catarrhalis*, *H. influenzae* and *F. necrophorum*). Across all test organisms, bactericidal activity was seen from the 1-min time point. For *S. aureus*, *M. catarrhalis*, *H. influenzae* and *F. necrophorum*, the decrease at 1 min exceeded 3log_10_ (99.9% reduction). For *S. pyogenes*, the most common cause of bacterial acute sore throat [3], the decrease was 2.9log_10_ at 1 min and greater than 3log_10_ reductions were recorded by 5 min. To the best of our knowledge, these are the first published data indicating that hexylresorcinol-containing lozenges have bactericidal activity in vitro.

The study was designed to simulate the clinical setting as far as possible, including the time taken for a lozenge to dissolve in the mouth (mean ± standard deviation: 6.77 ± 2.01 min) [22]. Furthermore, the method was designed to replicate the expected concentration of hexylresorcinol that would be achieved when a lozenge is dissolved in the mouth, assuming a volume of 5 mL of saliva.

The findings in this study are consistent with previously reported antibacterial effects of hexylresorcinol in solution against a range of organisms, including *Streptococcus spp.* and *S.* *aureus* [18]. In addition, these data are in line with studies of other over-the-counter acute sore throat treatments. In a similar in vitro study, Matthews et al. (2018) reported that lozenges containing 0.6 mg amylmetacresol and 1.2 mg 2,4-dichlorobenzyl alcohol had broad antibacterial activity against a similar range of oropharyngeal organisms associated with acute sore throat. Specifically, reductions in bacterial counts exceeded 99.9% by 1 min for *S. pyogenes*, *H. influenzae*, *F. necrophorum* and *A. haemolyticum*, by 5 min for *M. catarrhalis* and *S. dysgalactiae* and by 10 min for *S. aureus* [21]. The antibacterial effects of hexylresorcinol lozenges reported here add to existing knowledge of their activity, which include numbing effects [13, 14] and antiviral activity [16, 17], resulting in relief of sore throat symptoms [13].

In conclusion, hexylresorcinol lozenges demonstrated bactericidal activity against medically relevant oropharyngeal organisms associated with acute sore throat from 1 min and achieved a > 99.9% reduction in cfu/mL for all test organisms within 5 min, which is in line with the duration for a lozenge to dissolve in the mouth. Thus, hexylresorcinol lozenges represent an effective over-the-counter treatment option for acute sore throat, offering rapid antibacterial, antiviral and local anesthetic effects, and may help to avoid unnecessary antibiotic prescribing, which is associated with the development of antibiotic resistance.

## Limitations

- In vitro models cannot precisely replicate how lozenges will act in a patient’s throat. Therefore, additional studies may be needed to confirm the antibacterial activity of hexylresorcinol lozenges in a clinical setting.
- The lower limit of detection of the bactericidal activity assay in this study was rapidly reached following addition of hexylresorcinol: by 1-min exposure for *H. influenzae* and *F. necrophorum*, 5-min exposure for *S. aureus* and *M. catarrhalis* and 10-min exposure for *S. pyogenes*. The use of more sensitive analytical methods may have allowed for a greater limit of detection.

## Data Availability

The datasets used and/or analyzed during the current study are available from the corresponding author on reasonable request.

## Abbreviations

cfu: Colony-forming units
GABHS: Group A β-hemolytic Streptococcus
URTI: Upper respiratory tract infection

## References

[1] Pelucchi C, Grigoryan L, Galeone C, Esposito S, Huovinen P, Little P (2012). Guideline for the management of acute sore throat. Clin Microbiol Infect.

[2] Ebell MH, Smith MA, Barry HC, Ives K, Carey M (2000). The rational clinical examination. Does this patient have strep throat?. JAMA..

[3] Worrall GJ (2007). Acute sore throat. Can Fam Physician.

[4] Shephard A, Smith G, Aspley S, Schachtel BP (2015). Randomised, double-blind, placebo-controlled studies on flurbiprofen 8.75 mg lozenges in patients with/without group A or C streptococcal throat infection, with an assessment of clinicians’ prediction of ‘strep throat’. Int J Clin Pract..

[5] Radkova E, Burova N, Bychkova V, DeVito R (2017). Efficacy of flurbiprofen 8.75 mg delivered as a spray or lozenge in patients with sore throat due to upper respiratory tract infection: a randomized, non-inferiority trial in the Russian Federation. J Pain Res..

[6] Dagnelie CF, Touw-Otten FW, Kuyvenhoven MM, Rozenberg-Arska M, de Melker RA (1993). Bacterial flora in patients presenting with sore throat in Dutch general practice. Fam Pract.

[7] Gergova RT, Petrova G, Gergov S, Minchev P, Mitov I, Strateva T (2016). Microbiological features of upper respiratory tract infections in bulgarian children for the period 1998–2014. Balkan Med J..

[8] Mihancea N (1998). Frequency and distribution per species, biotypes, resistance to antibiotics and beta-lactamase production of the haemophils isolated from patients with respiratory diseases. Roum Arch Microbiol Immunol.

[9] Batty A, Wren MW (2005). Prevalence of Fusobacterium necrophorum and other upper respiratory tract pathogens isolated from throat swabs. Br J Biomed Sci.

[10] van Driel ML, De Sutter A, Deveugele M, Peersman W, Butler CC, De Meyere M (2006). Are sore throat patients who hope for antibiotics actually asking for pain relief?. Ann Fam Med..

[11] WHO (World Health Organization). Antibiotic resistance key facts. February 2018. https://www.who.int/en/news-room/fact-sheets/detail/antibiotic-resistance. Accessed 5 Feb 2020.

[12] Addey D, Shephard A (2012). Incidence, causes, severity and treatment of throat discomfort: a four-region online questionnaire survey. BMC Ear Nose Throat Disord..

[13] McNally D, Shephard A, Field E (2012). Randomised, double-blind, placebo-controlled study of a single dose of an amylmetacresol/2,4-dichlorobenzyl alcohol plus lidocaine lozenge or a hexylresorcinol lozenge for the treatment of acute sore throat due to upper respiratory tract infection. J Pharm Pharm Sci..

[14] McNally D and Scheiner M. Acute sore throat, Module 1551. April 2011. http://www.chemistanddruggist.co.uk/maincontent/-/article_display_list/4406146/updatemodule-1551-acute-sore-throat. Accessed 5 Feb 2020.

[15] Buchholz V, Leuwer M, Ahrens J, Foadi N, Krampfl K, Haeseler G (2009). Topical antiseptics for the treatment of sore throat block voltage-gated neuronal sodium channels in a local anaesthetic-like manner. Naunyn Schmiedebergs Arch Pharmacol..

[16] Shephard A, Zybeshari S (2015). Virucidal action of sore throat lozenges against respiratory viruses parainfluenza type 3 and cytomegalovirus. Antiviral Res.

[17] Morokutti-Kurz M, Graf C, Prieschl-Grassauer E (2017). Amylmetacresol/2,4-dichlorobenzyl alcohol, hexylresorcinol, or carrageenan lozenges as active treatments for sore throat. Int J Gen Med..

[18] Chaudhuri RK, Sivamani R, Jagdeo JR, Elsner P, Maibach HI (2015). Hexylresorcinol: Providing skin benefits by modulating multiple molecular targets. Cosmeceuticals and Active Cosmetics.

[19] Kemme M, Heinzel-Wieland R (2018). Quantitative assessment of antimicrobial activity of PLGA films loaded with 4-hexylresorcinol. J Funct Biomater..

[20] NIH (US National Library of Medicine). PubChem. Hexylresorcinol. https://pubchem.ncbi.nlm.nih.gov/compound/Hexylresorcinol. Accessed 5 Feb 2020.

[21] Matthews D, Atkinson R, Shephard A (2018). Spectrum of bactericidal action of amylmetacresol/2,4-dichlorobenzyl alcohol lozenges against oropharyngeal organisms implicated in pharyngitis. Int J Gen Med..

[22] Wade AG, Morris C, Shephard A, Crawford GM, Goulder MA (2011). A multicentre, randomised, double-blind, single-dose study assessing the efficacy of AMC/DCBA Warm lozenge or AMC/DCBA Cool lozenge in the relief of acute sore throat. BMC Fam Pract..

